# Modification of the Muscular Flap Operation for Amputation below the Knee-joint

**Published:** 1842-08-06

**Authors:** 


### ANALECTA.

### *Modification of the Muscular Flap Operation for Amputation below the Knee-joint.*

[We extract from the Provincial Medical Journal of July 2d, the following passages, from a lecture upon a case of amputation below the knee, by Thomas Green, Surgeon to St. Peter's Hospital, and Lecturer on Surgery at the School of Medicine, Bristol, England. The clinical history of the case after operation presents nothing of interest, and is therefore omitted. The last ligature came away from the stump, which at other points was healed and in good condition, on the forty-fifth day.]

Amongst others, the high authority of Sir C. Bell is in favour of the circular operation, on account of the evils found to occur during the operation itself. He states as an objection to the flap amputation, that the muscle is larger than the integuments, and that the gastrocnemius projects beyond the skin, when the flap is brought over the face of the stump; he also says that the vessels and nerves are divided obliquely. These disadvantages are precisely what I have found to occur in practice from the usual flap operation; the projection of the muscle beyond the skin in muscular persons, when the flap is turned up, I have found a great inconvenience, and one which makes it necessary to strain the integuments more than is desirable to bring them over the mass of muscle. One of the evils, and not the least, is the manner in which the nerves are sometimes divided; it will occasionally be found that the long knife, in passing downwards to form the posterior flap, carries along its edge for some distance the large nerve of the leg before it is divided by the movement of the instrument backwards; this I have found to occur in two cases in which I have operated at the hospital, when fully an inch of the posterior tibial nerve was in each case hanging out from the flap, rendering it necessary to cut the nerve off close to the surface. Some of you have seen the pain which this has caused, though done as quickly as possible; the vessels are also divided obliquely, and here I have found greater difficulty in securing them than when cut transversely. The practical inconveniences attendant on this mode of operating I consider to be three; first, the projection of the muscle beyond the integuments; second, the oblique division of nerves; and lastly, the oblique division of the vessels. These, I admit, are all serious objections to the usual flap operation at this place, and have been sufficient to induce Sir C. Bell to recommend its being given up altogether. The same difficulty has occurred to other surgeons during the past and present century, and various contrivances have at different times been thought of, and employed, to obviate these disadvantages, by those who, for other reasons, give a preference to the flap. It has been proposed to cut off a portion of the muscle from the flap; in fact, to slice off that which is superabundant. I need hardly say that this appears to be altogether objectionable. The German surgeons have tried different ways of diminishing the quantity of muscle while preserving sufficient integument to cover the flap—such as drawing up the skin, and bending the ankle-joint at the time the incision is being made. This proceeding would be likely, to some extent, to obviate the difficulty, but not, I should think, sufficiently so to remove it entirely; at least, not in muscular persons. A knife curved laterally was employed, I believe, by Graafe, with the intention of diminishing the quantity of muscle by scooping a portion of it from the flap as it was carried along. Of these contrivances I can say nothing from my own experience, but should consider them not sufficient to prevent entirely the evils I have spoken of.

The mode of operating employed in this case I first tried a few months ago at St. Peter's Hospital, on a man named Morris, and found that by it all the inconveniences alluded to were avoided, for the man left the house with an exceedingly good stump, and now makes good use of it in his vocation as a sweeper of one of the crossings at Clifton. I shall now describe the operation as I performed it yesterday, and if you examine the limb removed, which lies on the table, you will easily understand its stages. An incision was made anteriorly across the fore part of the leg, in the usual situation, about two inches below the tuberosity of the tibia; it extended from the inner angle of that bone to a point behind the fibula; from the termination of this incision, on the inner side of the leg, the knife was carried downwards to some extent, next across the limb posteriorly in a curved line, and brought up at the outer side, so as to unite with the front incision behind the fibula. In this manner a portion of integument was divided, which might be correctly described as representing two-thirds of an oval figure. This incision should go through the skin and subjacent tissue, down to the fascia covering the muscles; the contraction of the integument itself, with a trifling assistance, by drawing the skin upwards, leaving a separation of about half an inch between the edges of the incision. A long catlin was now pushed through the leg, about one-third of an inch behind the bones, and carried downwards, and next backwards, so as to make a flap of muscle, its edges corresponding with those of the retracted integument. The remaining muscles were next divided transversely; in this division are contained the large vessels and nerves, which are those [thus?] cut transversely. The bones were separated, the sharp angle of the tibia was sawn off, and the arteries secured in the usual way. The flap, when brought up over the face of the stump, was entirely and abundantly covered by skin; three sutures were used, assisted by two broad pieces of strapping; a cloth wetted in cold water was applied over the stump, and the man removed. I prefer sutures in this operation, on account of the weight of the flap having a tendency to draw it down, and thus separate it from the anterior surface. These I always remove on the third day, and have not found any inconvenience from their use.

